# Molecular Lysine Tweezers Counteract Aberrant Protein Aggregation

**DOI:** 10.3389/fchem.2019.00657

**Published:** 2019-10-01

**Authors:** Inesa Hadrovic, Philipp Rebmann, Frank-Gerrit Klärner, Gal Bitan, Thomas Schrader

**Affiliations:** ^1^Faculty of Chemistry, University of Duisburg-Essen, Essen, Germany; ^2^Department of Neurology, University of California, Los Angeles, Los Angeles, CA, United States

**Keywords:** molecular tweezers, amino acids, neurodegeneration, amyloids, protein aggregation

## Abstract

Molecular tweezers (MTs) are supramolecular host molecules equipped with two aromatic pincers linked together by a spacer (Gakh, 2018). They are endowed with fascinating properties originating from their ability to hold guests between their aromatic pincers (Chen and Whitlock, 1978; Zimmerman, 1991; Harmata, 2004). MTs are finding an increasing number of medicinal applications, e.g., as bis-intercalators for DNA such as the anticancer drug Ditercalinium (Gao et al., 1991), drug activity reverters such as the bisglycoluril tweezers Calabadion 1 (Ma et al., 2012) as well as radioimmuno detectors such as Venus flytrap clusters (Paxton et al., 1991). We recently embarked on a program to create water-soluble tweezers which selectively bind the side chains of lysine and arginine inside their cavity. This unique recognition mode is enabled by a torus-shaped, polycyclic framework, which is equipped with two hydrophilic phosphate groups. Cationic amino acid residues are bound by the synergistic effect of disperse, hydrophobic, and electrostatic interactions in a kinetically fast reversible process. Interactions of the same kind play a key role in numerous protein-protein interactions, as well as in pathologic protein aggregation. Therefore, these particular MTs show a high potential to disrupt such events, and indeed inhibit misfolding and self-assembly of amyloidogenic polypeptides without toxic side effects. The mini-review provides insight into the unique binding mode of MTs both toward peptides and aggregating proteins. It presents the synthesis of the lead compound CLR01 and its control, CLR03. Different biophysical experiments are explained which elucidate and help to better understand their mechanism of action. Specifically, we show how toxic aggregates of oligomeric and fibrillar protein species are dissolved and redirected to form amorphous, benign assemblies. Importantly, these new chemical tools are shown to be essentially non-toxic *in vivo*. Due to their reversible moderately tight binding, these agents are not protein-, but rather process-specific, which suggests a broad range of applications in protein misfolding events. Thus, MTs are highly promising candidates for disease-modifying therapy in early stages of neurodegenerative diseases. This is an outstanding example in the evolution of supramolecular concepts toward biological application.

## Introduction

A major challenge in modern medicine is the field of neurodegenerative diseases. Their pathology is dominated by misfolding and subsequent aggregation of characteristic peptides or proteins in the brain, which is correlated with severe impairment of cognitive functions. As the most prominent example, the amyloid β-peptide (Aβ) plays a key role in the development and progression of Alzheimer's disease (AD) (Hardy and Higgins, 1992; Hardy and Selkoe, 2002). Senile plaques composed of aggregated Aβ, forming extracellular β-sheet fibril morphologies, are histopathological hallmarks found in the brains of AD patients. In recent years however, small soluble Aβ oligomers were identified as the most neurotoxic species (Shankar et al., 2008; Zhao et al., 2012; Sengupta et al., 2016). Despite intense research, the underlying mechanisms of spontaneous misfolding, aggregation, and lesion of nerve cells are still poorly understood. To date only symptom-relieving drugs are clinically approved for AD treatment. Strategically, it seems desirable to develop drug candidates which are able to interfere with the early stages of the disease mechanism. Classical approaches include the reduction of Aβ production by inhibitors of β- and γ- secretase, the increase of Aβ removal via anti-Aβ immunotherapy, and direct interference with Aβ aggregation (Hardy and Selkoe, 2002; Roland and Jacobsen, 2009). The latter can be achieved with a diverse set of peptides and small molecules. Well-known milestones in this field are Congo red (Podlisny et al., 1998), scyllo-Inositol (McLaurin et al., 2000), amino-propane sulfonic acid (Gervais et al., 2007), Clioquinol (Cherny et al., 2001), methylene blue (Necula et al., 2007), and polyphenol (–)-epigallocatechin (EGCG) (Ehrnhoefer et al., 2008).

However, some of these compounds are toxic, others are only active in cell culture or animal experiments, and until today no drug candidate made it through clinical trial. In addition, little structural information is available about the direct interaction between Aβ and most aggregation inhibitors. Thus, there is clearly a need for new rational approaches. Supramolecular Chemistry has gained a much-improved understanding and quantitative description of those non-covalent interactions which are involved in protein aggregation. In addition, molecular modeling now allows extended MD simulations of complex ensembles with large sampling times and discrete solvent treatment—resulting in predictive power for new supramolecular binders. In our group we developed a highly selective host molecule for lysine and arginine, which is able to draw their side chains into its cavity and shield them from the environment. These molecular tweezers turned out to completely disrupt existing β-sheets formed by amyloidogenic proteins. Our discovery started an intense and very fruitful collaboration between supramolecular chemists and neurologists, which has reached the state of animal experiments and behavioral testing with transgenic mice and holds promise for the development of disease-modifying therapy. This mini-review summarizes the chemical aspects of the endeavor—from deciphering the binding mode of the tweezers over structural elucidation of their complexes with aggregating proteins to the characterization of their anti-aggregatory effect on various proteins. Finally, toxicity, metabolism, and bioavailability issues will also be briefly discussed.

## The Development of Molecular Tweezers as Lysine and Arginine Binders

### Structure and Binding Mode of Molecular Tweezers

**Figure 1:**
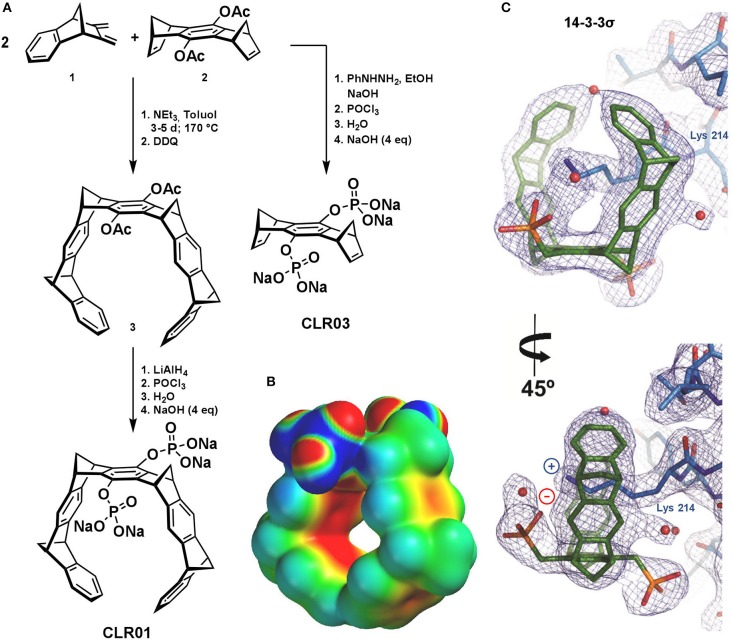
**(A)** Synthesis of the molecular tweezer CLR01 (Talbiersky et al., 2008) and negative control CLR03 (Kirsch et al., 2009). **(B)** Electrostatic Surface Potential of CLR01 calculated by PM3 implemented in SPARTAN 04 (Wavefunction Inc.) Color code spans from −25 kcal/mol (red) to +25 kcal/mol (blue), taken from (Heid et al., 2018, Figure 5). **(C)** 2Fo–Fc electron density (contoured at 1s) for the molecular tweezers bound to 14-3-3σ (Bier et al., 2013, Figure 4B).

There are numerous artificial binding motifs for naturally occurring amino acids, but only a few of them are selective and mild enough to find biological application (Crini, 2014; Barrow et al., 2015; Neri et al., 2016). Molecular tweezers were designed rationally, combining supramolecular knowledge, and total synthesis to obtain water-soluble horseshoe-shaped molecules. They are characterized by their well-preorganized torus-shaped, polycyclic non-polar framework, equipped with two hydrophilic phosphate groups. The uniqueness of MTs is reflected in their capability to selectively accommodate exclusively the side chains of basic amino acids, namely lysine and arginine, inside their cavity under physiological conditions. Electrostatic potential surface (EPS) calculations demonstrate that their cavity construction is electron-rich, perfectly symmetric, and open to receive cationic appropriately shaped guests (Figure 1B). It appears that even in PBS buffer tweezer dimerization is negligible (Dutt et al., 2013; Heid et al., 2018).

MTs bind cationic amino acid residues via threading their side chains through the cavity in a non-covalent fashion followed by formation of a salt bridge between the tweezer phosphate and the included ammonium or guanidinium cation. This is facilitated by exploiting in a synergistic way van der Waals interactions, substantial electrostatic contributions, and the non-classical hydrophobic effect; this binding mode results in a kinetically fast and reversible recognition process. Quantum chemical and molecular mechanics (QM/MM) calculations and various analytical experiments strongly support this postulated binding mode between MTs and their amino acid guests. Monte Carlo (MC) and molecular dynamics (MD) simulations, isothermal titration calorimetry (ITC) measurements, NMR, and fluorescence titrations, as well as NOESY and variable temperature (VT) experiments clearly point to inclusion of the lysine and arginine side chain inside the tweezer cavity in an enthalpy-driven process. QM/MM calculations confirm the existence of these favorable host-guest complexes in buffered aqueous solution formed via the threading mode (Fokkens et al., 2005; Dutt et al., 2013).

Recently, a crystal structure of the complex between MT and a 14-3-3 protein beautifully demonstrated the threading of the well accessible Lys-214 side chain through the tweezers' cavity accompanied in solution with a substantial inhibition of the complex formation between the 14-3-3 and its natural cargo proteins (Bier et al., 2013).

### Tweezer Synthesis

The characteristic MT framework consists of nine annulated 6-membered rings, which are alternating phenyl and norbornadiene ring systems. The construction of this hydrocarbon torus is achieved in the key step via double Diels-Alder (DA) cycloaddition using two equivalents of diene **1** which forms the walls and one equivalent of dienophile **2** which is the center-piece (Figure 1A). The exocyclic diene is obtained in six steps from indene and maleic acid anhydride while the dienophile is made from 1,4-benzophenone in a four-step sequence. The neutral DA reaction requires elevated temperatures; it proceeds stereoselectively *endo* in **1** and *exo* in **2** and thus leads after DDQ (2,3-dichloro-5,6-dicyano-1,4-benzoquinone) oxidation to the desired tweezer (**3**) having the four methylene bridges in all-*syn* configuration (Klärner et al., 1996, 1999; Talbiersky et al., 2008; Schrader et al., 2016). The two acetoxy-groups can be cleaved in a symmetric or asymmetric fashion, releasing hydroquinone OH groups which can be further functionalized with negatively charged groups for enhanced water-solubility (e.g., phosphates, carboxylates, and sulfates) (Dutt et al., 2013). In the course of several years of intense biophysical and biological testing, the tweezers CLR01 with its two phosphate esters evolved as a lead compound, while its truncated derivative without the side walls, CLR03, served as a negative control. CLR03 represents the central part of the MT molecule; due to the lack of the torus-shaped cavity, it is not able to bind Lys and Arg by inclusion (Schrader et al., 2016).

## Interaction With Bioactive Peptides

CLR01 was initially tested with small, biologically relevant small peptides (Fokkens et al., 2005). The KLVFF peptide is located inside the central hydrophobic part of the amyloid-β protein, and it was identified as a nucleation site for pathologic protein aggregation, fibril formation, and subsequent plaque occurrence in Alzheimer's disease. NMR and fluorescence titrations with this small peptide revealed inclusion of the *N-*terminal lysine inside CLR01 and a moderate affinity of 10 μM (*K*_d_) in buffered aqueous solution (PBS) (Dutt et al., 2013).

ITC measurements provide further insight into the postulated binding mode. The binding event between host CLR01 and its KLVFF guest was shown to be a favorable, strongly exothermic process. Here the MT peptide affinity was found to be 15 μM, with a 1:1 stoichiometry, and an enthalpic contribution Δ*H* of −6.6 kcal/mol, which is prevailing over the small entropy term –*T*Δ*S* of −0.2 kcal/mol. Arginine complexation in other peptides was found to be slightly weaker, in the range of 30 μM, most likely due to its delocalized guanidinium ion and shorter side chain. The remarkably exothermic character of the binding event correlates well with the assumed threading procedure and the resulting van der Waals interactions between the host cavity and the respective amino acid side chain. The above-reported *K*_d_ values, although moderate in biological terms, place these MT among the most efficient receptor molecules for basic amino acids known today (Fokkens et al., 2005; Dutt et al., 2013).

In general, dissociation constants obtained from ITC measurements agree well with the data determined independently by fluorescence or ^1^H NMR titrations, in spite of the different concentration regimes (NMR 10^−3^ M, ITC 10^−4^ M, Fluoresc. 10^−5^ M). The emission intensity maximum of MTs in fluorescence spectra is found around 330 nm, while the excitation maximum is located at 285 nm (π,π^*^). Trapping of guest molecules inside the tweezers cavity results in significant quenching of the fluorescence emission. This proves guest inclusion and allows quantification of the binding event at low concentrations. In most cases affinities for a single lysine inclusion determined by fluorometric titrations are in the range of 5–20 μM *K*_d_. Structurally, the MT's preference for lysine inclusion has been proven in numerous ^1^H NMR titrations in buffer, which reveal drastic upfield shifts of up to 4 ppm (δΔ_max_) at the δ*-* and ε*-* methylene protons of the basic amino acid side chains. NOESY measurements as well as variable temperature experiments strongly support the guest inclusion (Fokkens et al., 2005).

Molecular tweezers with their unique binding mode for lysine and arginine and their unexpected powerful effect as aggregation inhibitors have attracted the attention of many research groups worldwide in the last decade. Numerous fruitful collaborations demonstrated that these lysine binders represent a widely applicable useful tool against pathologic protein misfolding. In addition, sophisticated analytical methods opened our understanding of the underlying supramolecular mechanism of action. Today we know that advanced MTs are able to specifically disrupt undesired protein-protein interactions; however perhaps even more important is the fact that MTs indeed inhibit misfolding and self-assembly of amyloidogenic polypeptides without toxic side effects (Sinha et al., 2011).

## Interaction Between Molecular Tweezers and Aggregating Proteins

The pathogenesis of every amyloidosis is caused by aberrant protein aggregation and most likely begins with protein misfolding. AD, Parkinson's disease and type-2 diabetes are the best examined examples of this pathologic process. In the course of AD, the largely unstructured naturally occurring monomeric state of the amyloid-β peptides was shown to adopt a conformation rich in β-sheets and which aberrantly forms toxic oligomers and aggregates (Billings et al., 2005). Aβ40, Aβ42 and the group of tau proteins mainly participate in this neurologically highly relevant aggregation process which ultimately disposes extracellular plaque formed from β-sheet-rich fibrils. Lysine residues are reported to play an important role in this particular assembly (Usui et al., 2009; Sinha et al., 2012).

Gratifyingly, MT were found to interfere with the aggregation process of most amyloidogenic proteins. In recent years, many different experiments have been designed and conducted which confirmed CLR01 to be capable of dissolving fibrils, preventing their formation as well as eliminating their toxic precursor oligomers. Structurally, it was important to identify the tweezer binding sites on these proteins. For the most prominent representative, the Alzheimer's peptide, the preferred complexation sites of the tweezers were validated by electron capture dissociation (ECD) mass experiments (EDC-MS/MS) as well as by NMR spectroscopy (Sinha et al., 2011).

### ECD-MS/MS

In the monomeric form of Aβ40 and Aβ42 there are three basic residues, Arg-5, Lys-16, and Lys-28. All of these are simultaneously complexed as confirmed by mass spectrometry. In EDC-MS/MS experiments complexes of MT and a Aβ protein were collected in a linear ion trap and smoothly fragmented inside. The recorded MS spectra found MT bound to many overlapping protein fragments. The mass spectra show peaks for Aβ40 bound by one, two and three MTs, respectively. In the fragmentation pattern CLR01-Aβ-fragment peaks were only found for fragments bearing a Lys or Arg residue, indicating a retained amino acid selectivity in Aβ complexation. Most importantly, peptide cleavage did not occur around the two lysine binding sites, because these were protected by the tweezers. The exact binding mode of this complexation event was subsequently investigated by NMR experiments (Sinha et al., 2011).

### NMR Experiments

**Figure 2:**
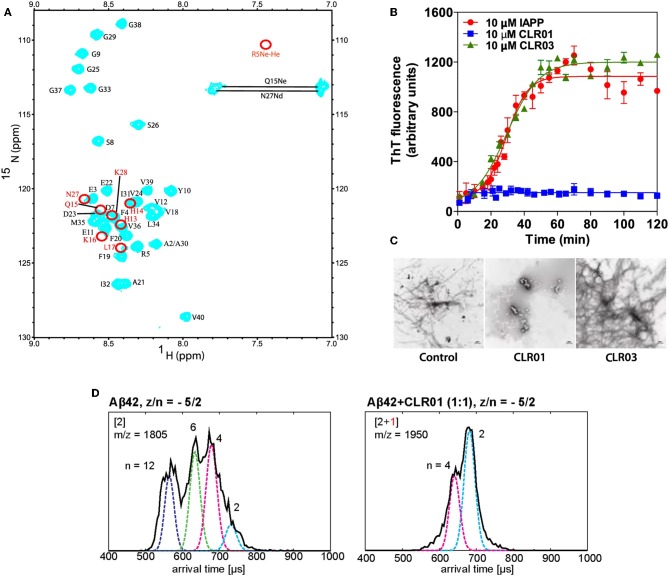
**(A)** ^15^N–^1^H HSQC spectrum of Aβ40 in the presence of 30 μM CLR01 (cyan). Red circles indicate resonances that disappeared completely upon addition of CLR01 to Aβ40 solution (Sinha et al., 2011). **(B)** Time-dependent ThT fluorescence intensities of incubated solution of: pure IAPP (red); IAPP + CLR01 (1:1) (blue); IAPP+CLR03 (1:1) (green) (Sinha et al., 2011). **(C)** EM images of the solutions after titration. CLR01 (middle) clearly shows inhibition of fibril formation (Sinha et al., 2011). **(D)** Effects CLR01 on Aβ42 early oligomerization. Arrival time distributions (by ion-mobility spectrometry) of z/n = −5/2 Aβ42 (m/z = 1805) in an Aβ42 sample without CLR01 (left) and z/n = −5/2 Aβ42 and CLR01 1:1-complex (m/z = 1,950). Oligomeric species (*n* = 6;12) vanish after CLR01 addition (Zheng et al., 2015).

^1^H–^15^N and ^1^H–^13^C heteronuclear single quantum coherence (HSQC) NMR experiments confirmed these results. An HSQC spectrum of Aβ40 alone and together with 0.5 equivalents of MT were compared. Upon tweezer binding, the cross peaks of the complexed residue as well as its neighboring amino acids show a significant chemical shift perturbation (CSP) due to the altered magnetic environment. Some signals vanished completely, indicated by red circles in Figure 2A. In two-dimensional H(N)CO experiments MT were titrated to a protein solution; already at a 1:10 ratio of CLR01 relative to Aβ40 the CSP became significant. Amino acid residues surrounding Lys-16 and Lys-28 showed a higher degree of perturbation compared to those in proximity to Arg-5. This implies a stronger affinity of MT for the Aβ Lys residues, consistent with the general Lysine preference of CLR01. At elevated CLR01 concentration all three positions were occupied. The negative control CLR03 showed no effects in the whole NMR-setup (Sinha et al., 2011). Similarly, a three-dimensional HN(CO)CACB NMR experiment was recently used to detect binding sites of MT in the phosphorylated and unphosphorylated tau protein (Despres et al., 2019).

The combination of EDC-MS/MS and NMR spectroscopy underlines the importance of the tweezer cavity for complex formation with Aβ40 and Aβ42, and strongly supports the binding mode elucidated with small peptides (Figure 1C).

If CLR01 binds to every sterically accessible basic residue on a peptide or protein, why is it not toxic then? It was indeed shown that the MT are non-toxic in biological applications at concentrations necessary to inhibit protein aggregation. We believe, that the key lies in kinetically fast reversible binding and moderate affinity. Biophysical experiments indicate fast on and off rates and labile complexation as well as moderate dissociation constants in the low μM range (Talbiersky et al., 2008; Bier et al., 2013). These key features of our MT safeguard healthy proteins from damage induced by conformational changes, so that they retain their natural biological function. Indeed, enzymes could be inhibited by MT, albeit only at millimolar concentrations, 100 times higher than those required for the anti-aggregatory effect. The specificity of CLR01 toward the process of aberrant protein aggregation is outstanding because it is a new principle which may be transferred to other drugs as well. It appears that MTs bind their protein guests with the same combination of non-covalent interactions which is also active in the unwanted aggregation process. This unique way of action toward protein aggregation, represents the first example of a “process-specific” aggregation inhibitor; it was examined with various biophysical methods (Schrader et al., 2016).

## Prevention of Pathologic Protein Aggregation

Until 2011 experimental evidence was accumulated for the fact that lysine-specific MT are active against a wide range of aggregating proteins, with detailed experiments performed on the assembly and toxicity of nine prominent amyloid proteins (Sinha et al., 2011).

In this comprehensive investigation a synopsis of various state-of-the-art biophysical experiments gave the full picture: Thioflavin T (ThT) fluorescence, Electron Microscopy (EM), Circular Dichroism spectroscopy (CD), Dynamic Light Scattering (DLS), Mass Spectrometry (MS), and NMR Spectroscopy.

### Thioflavin T (ThT) Fluorescence

ThT is an amyloid dye indicator which turns highly fluorescent upon binding to existing β-sheets (LeVine, 1999). ThT fluorescence was used to monitor the kinetics of β-sheet formation for various amyloid proteins in the presence or absence of CLR01. The tweezers represented the active drug, whereas their truncated derivative, CLR03, was used as negative control. Measurements were performed regularly during a time span of up to 120 h at pH 7.4 in 10 mM phosphate buffer. CLR01 was added in up to 10-fold excess relative to the protein and was able to completely suppress the typical drastic fluorescence enhancement caused by aggregation and protein misfolding. Equimolar concentration of CLR01 was likewise shown to totally disrupt β-sheets of the tau protein. CLR03 displayed no effect in any of the investigated proteins because it lacks the hydrophobic side walls and consequently, the ability to complex Lys residues. Importantly, CLR01 not only inhibited the *de novo* aggregation of amyloidogenic proteins such as Aβ40/Aβ42, α-synuclein and IAPP (Figure 2B) (Prabhudesai et al., 2012), but also disaggregated pre-formed fibrils over several weeks when added at a 10-fold excess, as being confirmed by EM.

### Electron Microscopy (EM)

EM measurements were carried out in parallel to ThT assays, by spotting 10 μL aliquots taken from each aggregation reaction, on glow discharged, carbon-coated Formvar grids, using a CX 100 transmission electron microscope. Visualization of the protein morphology showed that IAPP and other examined amyloidogenic protein samples incubated in the presence of MTs did not form fibrils anymore, strongly supporting conclusions drawn from the ThT measurements (Figure 2C) (Sinha et al., 2011).

### CD Spectroscopy

All β-sheets and therefore also all pathologic protein aggregates produce a dominant characteristic β-sheet band at 215 nm in the CD spectrum. In the presence of a 3-fold excess of CLR01, this band was rapidly reduced and completely disappeared after 1 h, indicating efficient inhibition of β*-*sheet formation in case of Aβ40 and Aβ42. Equimolar CLR01 lead to partial inhibition. Interestingly, CLR01 completely inhibited tau aggregation already at the equimolar level, which correlates with the higher number of exposed Lys residues in the tau sequence in comparison to Aβ (Sinha et al., 2011).

### Dynamic Light Scattering (DLS)

Dynamic light scattering provides a direct and non-invasive way to monitor the formation of larger aggregates. It was employed to monitor the influence of CLR01 on oligomer size and distribution of Aβ. Experiments were performed with CLR01 in 10-fold excess or equimolar relative to Aβ, controls were run with Aβ alone. Intriguingly, the DLS results indicate that CLR01 does not prevent oligomer formation but rather modulates Aß self-assembly into formation of structures that are neither amyloidogenic nor toxic (Sinha et al., 2011).

### Mass Spectrometry (MS)

In recent years, advanced methods in mass spectrometry have been exploited for the mechanistic elucidation of protein aggregating events. Thus, Bowers et al. used mild ionization conditions and high resolution to monitor the impact of small molecule modulators on Aβ oligomerization (Zheng et al., 2015). The effect of different concentrations of CLR01 and its related derivate, CLR03 on the Aβ assembly was investigated with a custom-built ion mobility spectrometry-mass spectrometer (IMS-MS) which consisted of a nano electrospray ionization (nano-ESI) source, an ion funnel, a temperature-controlled drift cell, and a quadrupole mass filter followed by an electron multiplier for ion detection (Wyttenbach et al., 2001). Consistent with earlier studies (Sinha et al., 2011), these experiments confirmed Arg-5, Lys-16, and Lys-28 as preferred binding sites for MT on Aβ. The authors associated four distinct peaks with Aβ42 alone, while in the presence of a 10-fold CLR01-excess three sets of peaks occurred corresponding to different charge states of the complexes of Aβ42 with up to four bound tweezers (Bernstein et al., 2009). No dimers or higher oligomers were observed (Figure 2D). This is a good indication that CLR01 not only prevents formation of Aβ42 dimers, but also of higher order oligomers. Importantly, no free, unbound Aβ42 was found in the mass spectrum, supporting the assumption that MT bind directly to Aβ42 with rather high affinity (~ 1 μM). The authors concluded that CLR01 can remodel the early oligomerization of Aβ42 not only immediately upon dissolution but also after the oligomers have already been formed (Zheng et al., 2015).

Native Top-Down Mass Spectrometry and IMS were likewise used to characterize the interaction between MT and the Tau Protein (Nshanian et al., 2018).

Very recently, Loo also reported that no toxic oligomers are left as the result of the efficient interaction between MT and SOD1 (Superoxide Dismutase 1). With ECD, the covalent peptide bonds of the polypeptide could be cleaved, whereas non-covalent forces sufficed to hold the ligand bound to the macromolecule. Tandem MS (MS/MS) or “top-down” MS of the protein–ligand complex allowed to explore the main binding site(s) of MT on the SOD1 surface. Surprisingly, MT preferred to bind to Lys-70 and/or Lys-75 although none of these residues is directly involved in the aggregation process of SOD1. This may explain why at least a 5-fold MT excess is required to affect the aggregation (Malik et al., 2019).

In this investigation, CLR01 inhibited abnormal SOD1 self-assembly *in vitro*, as well as *in vivo*, as being shown on the G93A-SOD1 mouse model of amyotrophic lateral sclerosis (ALS). By applying therapeutic amounts of CLR01 to recombinant wild type and mutant SOD1, their *in vitro* aggregation speed was significantly lowered for all SOD1 forms. *In vivo*, misfolded SOD1 in the spinal cord was significantly reduced, yet not enough to overcome motor deficits, most likely due to the fast disease progression. Further insight came from experiments on SOD1 with ThT and EM at the end of each aggregation. For a potential SOD1 treatment, advanced tweezer derivatives with improved performance must be designed in the future (Malik et al., 2019).

## Conclusion and Outlook

The above-discussed synopsis of structural and biophysical experiments strongly suggests that molecular tweezers dock onto sterically accessible lysine and arginine on aggregating proteins. The resulting Lys/Arg shielding prevents misfolding and/or subsequent protein aggregation into toxic oligomers. It also dissolves existing β-sheets and redirects their path of aggregation to benign amorphous structures.

The same effects are observed with a large number of aggregating proteins, so that these lysine binders seem to act in a process-specific manner and are clearly not protein-specific. In all cases where lysine or arginine residues are involved in the aggregation process, molecular tweezers seem to prevent their ordered aggregation into fibrillar toxic structures. It may be argued that unselective multiple lysine binding should greatly disturb protein function; however, their moderate affinity (10–30 μM K_d_) and fast on- and off-rates apparently preserve natural protein folding and function. Indeed, for enzyme inhibition 100-fold higher concentrations are needed (1 mM), providing a large potential therapeutic window.

After the initial aggregation assays with isolated proteins, cell culture experiments demonstrated powerful protection against oligomer or fibril lesion from exactly those proteins whose aggregation could also be rescued *in vitro* (Xu et al., 2017; Malik et al., 2018). Finally, triply transgenic mice were treated with low daily doses of CLR01 (40 μg/kg) and showed dramatic reduction of plaque load in their cortices (stained histological brain slices). Subsequent behavioral tests (Y-Maze, Pole climbing) revealed significant memory and mobility improvement after treatment with CLR01 (Richter et al., 2017).

Although no systematic metabolism studies have been carried out yet, no degradation product could be found so far, e.g., after treatment with strong acid (pH 0) and base (pH 12) and common phosphatases. We assume that the steric demand of the tweezer skeleton prevents most chemical transformations at the two phosphate groups, and that the doubly phosphorylated stage is recognized as a water-soluble metabolite ready for urinary excretion.

CLR01 was tritium-labeled and could be detected in mouse brains. In addition, HPLC-MS assays of brain extracts revealed 2-3 nM concentrations of CLR01. We are currently optimizing the tweezer structure to generate aggregation inhibitors of lower polarity which will cross the blood-brain barrier (BBB) much more efficiently, hopefully even after oral administration. In some of these projects the results are so promising that we hope to enter clinical trial within the next few years.

Thus, a supramolecular host molecule for basic amino acids was turned into a powerful tool against pathologic protein aggregation and showed highly promising effects in various cell types and animal models. This is an outstanding example in the evolution of supramolecular concepts toward biological application.

## Author Contributions

PR wrote the chapters about structure, synthesis, and binding of MTs. IH wrote the chapters about prevention of pathologic protein aggregation by MTs. TS wrote the introduction and conclusions section. F-GK invented the molecular tweezers, GB carried out biological experiments, and both supervised the revision of the mini-review.

### Conflict of Interest

The authors declare that the research was conducted in the absence of any commercial or financial relationships that could be construed as a potential conflict of interest.
